# Flavonoids Extracted from Licorice Prevents Colitis-Associated Carcinogenesis in AOM/DSS Mouse Model

**DOI:** 10.3390/ijms17091343

**Published:** 2016-08-24

**Authors:** Xiaowei Huo, Dongyu Liu, Li Gao, Liyong Li, Li Cao

**Affiliations:** Institute of Medicinal Plant Development, Chinese Academy of Medical Sciences and Peking Union Medical College, Beijing 100193, China; huoxiaoweiforever@163.com (X.H.); liuermengmeng@126.com (D.L.); mmliu.bio@gmail.com (L.G.); wangkun000guai@163.com (L.L.)

**Keywords:** licorice flavonoids, colitis-associated carcinogenesis, inflammatory bowel disease

## Abstract

Inflammatory bowel disease (IBD) is generally considered as a major risk factor in the progression of colitis-associated carcinogenesis (CAC). Thus, it is well accepted that ameliorating inflammation creates a potential to achieve an inhibitory effect on CAC. Licorice flavonoids (LFs) possess strong anti-inflammatory activity, making it possible to investigate its pharmacologic role in suppressing CAC. The purpose of the present study was to evaluate the anti-tumor potential of LFs, and further explore the underlying mechanisms. Firstly, an azoxymethane (AOM)/dextran sulfate sodium (DSS)-induced mouse model was established and administered with or without LFs for 10 weeks, and then the severity of CAC was examined macroscopically and histologically. Subsequently, the effects of LFs on expression of proteins associated with apoptosis and proliferation, levels of inflammatory cytokine, expression of phosphorylated-Janus kinases 2 (p-Jak2) and phosphorylated-signal transducer and activator of transcription 3 (p-Stat3), and activation of nuclear factor-κB (NFκB) and P53 were assessed. We found that LFs could significantly reduce tumorigenesis induced by AOM/DSS. Further study revealed that LFs treatment substantially reduced activation of NFκB and P53, and subsequently suppressed production of inflammatory cytokines and phosphorylation of Jak2 and Stat3 in AOM/DSS-induced mice. Taken together, LFs treatment alleviated AOM/DSS induced CAC via P53 and NFκB/IL-6/Jak2/Stat3 pathways, highlighting the potential of LFs in preventing CAC.

## 1. Introduction

Colitis-Associated Carcinogenesis (CAC) ranks the third most frequently diagnosed cancer and the second leading cause of cancer death in developed countries [1,2]. Inflammatory bowel disease (IBD), known as a group of disorders characterized by recurring inflammation of the lower intestine, is considered as an important factor in the progression of CAC, emphasizing that patients suffering from IBD, such as Crohn’s disease and Ulcerative colitis (UC), are at a high risk for CAC [3,4]. Uncontrolled expression of pro-inflammatory cytokines, chemokines, and growth factors is reported to underlie the chronic inflammatory state found in IBD [5]. Although the precise mechanism of action underlying development of CAC remains incompletely understood, pro-inflammatory cytokines and mediators produced during chronic inflammation in IBD are thought to implicated in CAC through their capacity to activate Janus kinases 2 (Jak2), and signal transducer and activator of transcription 3 (Stat3), thereby contributing to development of neoplastic cells transformed from colonic epithelial cells [6,7]. Therefore, it is anticipated that agents capable of ameliorating inflammation may control progression of CAC, creating a potential to achieve a great cancer inhibitory benefit.

In fact, non-steroidal anti-inflammatory drugs (NSAIDS) can reduce IBD-related CAC formation, however, a series of concerns and long-term risks of this form chemoprevention make them unsuitable as a general recommendation for CAC treatment [8]. Given the limitations of today’s standards of practice in prevention of colorectal tumorigenesis, it is essential to explore alternative strategies for colitis and colorectal tumorigenesis.

Licorice, the roots and stolons of *Glycyrrhiza* species, is a traditional Chinese herbal medicine that has been used for a long history in many Asian countries for different medical purposes [9,10,11]. Owing to its sweet taste, it is also used worldwide in food products as a sweetening and flavoring component [12,13]. Licorice is rich in flavonoids and triterpenoids, among which the flavonoids isolated from licorice have attained a considerable interest for the diversity of their chemical structures and biological activities [14]. Previous investigations, of active components in licorice, mainly focused on LFs, as they were considered to be responsible for anti-inflammatory effects [15,16]. Moreover, the anti-inflammatory effects of LFs have been demonstrated recently by our group in DSS induced UC [17], thus, it is rational to speculated that their pharmacological properties may be applicable for the treatment of CAC.

The purpose of the present study is to verify the anti-colorectal cancer potential of LFs, and further explore the underlying mechanisms. We choose an AOM/DSS mouse model, in which the colonic inflammation was induced to mimic progression of CAC observed in humans. Our findings demonstrated that LFs could significantly inhibit AOM/DSS induced inflammation and tumorigenesis, involving a mechanism of blocking P53 and NFκB/IL-6/Jak2/Stat3 pathways, indicating that LFs has potential for the suppression of CAC.

## 2. Results

### 2.1. LFs Increased the Survival Rate of AOM/DSS Induced Mice

**Figure 1:**
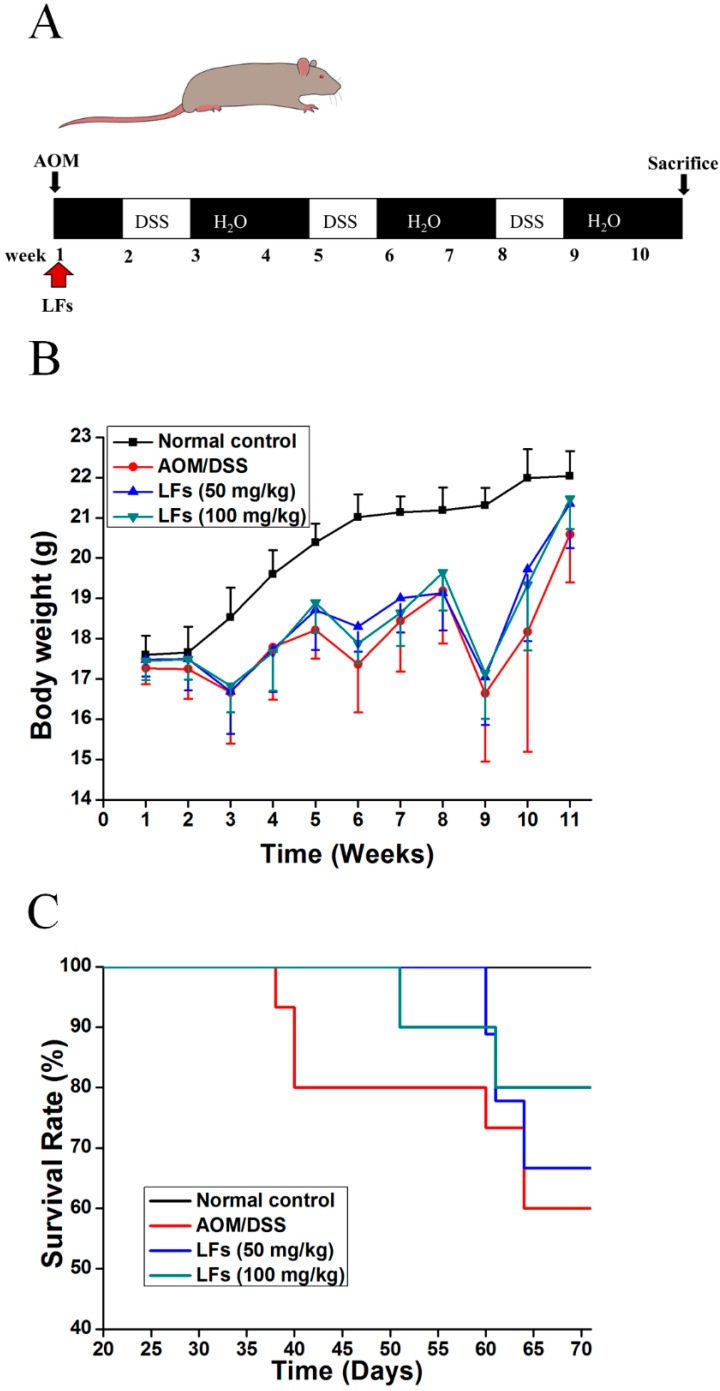
Effects of LFs on colitis-associated colon carcinogenesis were evaluated in C57BL/6 mice. (**A**) Schematic of administration of AOM, DSS and LFs to mice. Fifteen mice were set in the model control group, and 10 mice per group were set in other groups; (**B**) Effect of LFs on the body weight of mice. During experiment, mice were weighed once a week for 10 weeks; (**C**) Effect of LFs on the survival rate of mice. LFs prolonged animal survival. Data are presented as mean vs. control and model.

To examine the effect of LFs on CAC, mutagen AOM was used to initiate colon tumors, followed by repeated DSS administration to induce chronic inflammation (Figure 1A). Mice receiving vehicle alone were used as a control group. Throughout the AOM/DSS treatment, mice were orally administrated with LFs (0, 50, and 100 mg/kg) once a day for 10 weeks. As shown in Figure 1B, significant body weight loss was observed in AOM/DSS induced mice when compared with the control group, which appeared to be alleviated by LFs treatment but this was not significant. Moreover, the survival rate of AOM/DSS induced mice was significantly increased after LFs treatment (50 and 100 mg/kg) based on Kaplan–Meier survival curves (Figure 1C), with survival rates of 66% and 80% at the end of the experiment, respectively.

### 2.2. LFs Suppresses Colitis-Associated Colon Tumorigenesis

**Figure 2:**
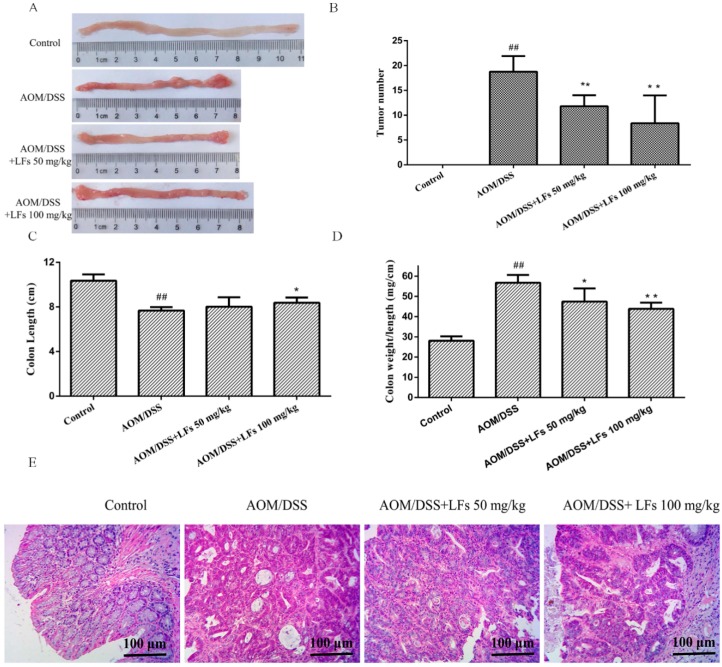
Effects of LFs on the burden of colonic neoplasms. (**A**) Microscopic view of colon in mice. AOM/DSS treatment resulted in 100% incidence of colonic neoplasms and no neoplasm was observed in control group; (**B**) LFs treatment reduced numbers of colonic neoplasms. Data are presented as mean ± SD. ** *p* < 0.01 vs. model, ^##^ *p* < 0.01 vs. vehicle control; (**C**) Effect of LFs on colon length. Reduced colon length induced by azoxymethane (AOM)/dextran sulfate sodium (DSS) was significantly reversed by LFs treatment. Data are presented as mean ± SD. * *p* < 0.05 vs. model, ^##^ *p* < 0.01 vs. vehicle control; (**D**) Effects of LFs on colon weight to colon length ratio. Colon weight to colon length ratio was assessed after mice were treated with LFs for 10 weeks. Data are presented as mean ± SD. * *p* < 0.05, ** *p* < 0.01 vs. model, ^##^ *p* < 0.01 vs. vehicle control; (**E**) Hematoxylin and eosin (H&E) staining of colons. Histological studies were conducted through hematoxylin and eosin staining. Most colorectal neoplasms were histologically determined as adenoma.

Tumor formation was analyzed at the end of the experiment. As shown in Figure 2A,B, in the absence of LFs treatment, AOM/DSS-induced mice exhibited a high tumor burden in the colons, while LFs treatment markedly reduced AOM/DSS-induced tumors. Moreover, decreased colon length was observed in AOM/DSS-induced mice, when compared with the control mice. Such significant decrease was relieved by LFs treatment at 100 mg/kg (Figure 2C). In addition, AOM/DSS treatment could significantly increase colon weight to colon length ratio when compared with mice treated with vehicle control (Figure 2D), which seemed to be a result of apparent mucosal thickening, and LFs treatment significantly decrease this ratio suggesting substantial alleviation of inflammation. Hematoxylin and eosin (H&E) staining of colon tissues was performed in order to analyze the pathology of AOM/DSS-induced colons. The result showed that LFs greatly suppressed the development of CAC induced by AOM/DSS treatment (Figure 2E). These data indicate that LFs exhibits strong suppressive effect on colitis and colorectal tumorigenesis induced by AOM/DSS.

### 2.3. LFs Regulated Proteins Associated with Apoptosis and Proliferation in Colonic Tissues

**Figure 3:**
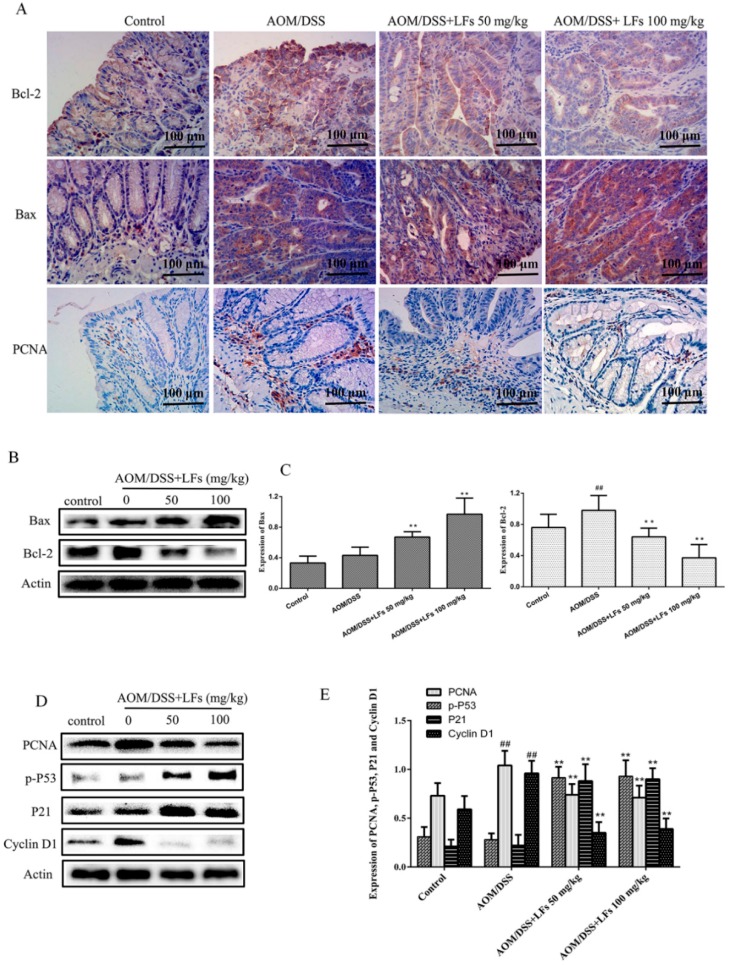
Effects of LFs on expression of proteins associated with proliferation and apoptosis in colonic tissues: (**A**) Immunohistochemical staining of Bax, Bcl-2, and PCNA in colonic tissues; (**B**,**C**) Western blot of Bax and Bcl-2 expression in colonic tissues and semi-quantitative analysis of these proteins. Data are presented as mean ± SD. ** *p* < 0.01 vs. model, ^##^ *p* < 0.01 vs. vehicle control; (**D**,**E**) Western blot of PCNA, p-P53, P21, and CyclinD1 expression in colonic tissues and semi-quantitative analysis of these proteins. Data are presented as mean ± SD. ** *p* < 0.01 vs. model, ^##^ *p* < 0.01 vs. vehicle control.

Uncontrolled proliferation and evasion of apoptosis are considered common events during colon carcinogenesis. Accordingly, we examined protein levels of Bax and Bcl-2, corresponding to apoptosis and expression of proliferating cell nuclear antigen (PCNA), p-P53, P21, and CyclinD1, corresponding to proliferation. IHC analysis demonstrated that the expression of pro-apoptosis protein Bax was elevated in LFs treated group; meanwhile, anti-apoptosis protein Bcl-2 was reduced by LFs treatment (Figure 3A). Western blot analysis confirmed that LFs treatment could substantially increase expression of Bax and reduce levels of Bcl-2 (Figure 3B,C), thus inducing apoptosis of tumors.

Next, epithelial proliferation was evaluated using PCNA staining and Western blot analysis. We noticed that there was a marked increase of PCNA expression in the colons of AOM/DSS induced mice, such increases were substantially reduced after LFs treatment (Figure 3A,D,E). Moreover, we found that the expression of p-P53 and P21, which were strongly linked to cell cycle arrest, in AOM/DSS-treated colons were greatly increased by LFs treatment (Figure 3D,E). LFs treatment also decreased the epithelial proliferation in AOM/DSS-treated mice by decreasing expression of CyclinD1, known as downstream of P53 and P21 (Figure 3D,E). These results indicate that LFs induce apoptosis and, meanwhile, reduced proliferation of epithelial cells in AOM/DSS-treated colons.

### 2.4. LFs Treatment Reduced Pro-Inflammatory Cytokines and Mediators

**Figure 4:**
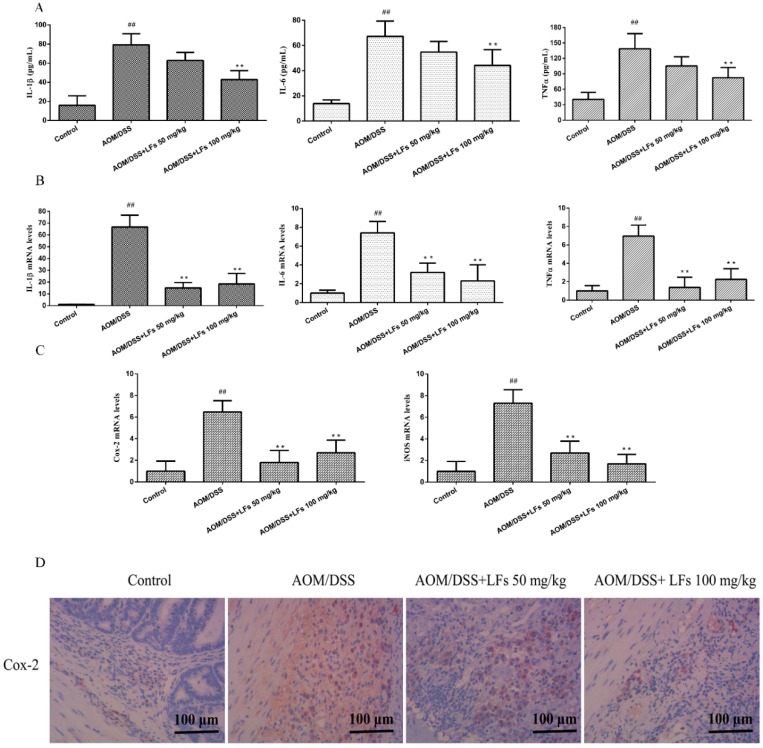
Effects of LFs on pro-inflammatory cytokines and mediators. (**A**) Interleukin-1β (IL-1β), Interleukin-6 (IL-6), and Tumor Necrosis Factor-α (TNF-α) levels in serum. ELISA kits specific to each cytokine were used to detected levels of IL-1β, IL-6, and TNF-α in serum. Data are presented as mean ± SD. ** *p* < 0.01 vs. model, ^##^ *p* < 0.01 vs. vehicle control; (**B**) mRNA levels of IL-1β, IL-6, and TNF-α in the colonic tissues. qRT-PCR analysis was carried out to evaluate mRNA levels of IL-1β, IL-6, and TNF-α. Data are presented as mean ± SD. ** *p* < 0.01 vs. model, ^##^ *p* < 0.01 vs. vehicle control; (**C**) mRNA levels of cyclooxygenase-2 (Cox-2) and inducible nitric oxide synthase (iNOS) in the colonic tissues by qRT-PCR analysis. Data are presented as mean ± SD. ** *p* < 0.01 vs. model, ^##^ *p* < 0.01 vs. vehicle control. (**D**) Immunohistochemical staining of Cox-2 in colonic tissues.

Chronic inflammation is now widely accepted as a promoter of colitis-associated colon carcinogenesis. We therefore evaluated whether the anti-tumor activity of LFs was associated with its anti-inflammatory properties through inhibiting pro-inflammatory cytokines and mediators (IL-1β, IL-6, TNF-α, iNOS and Cox-2). As shown in Figure 4A, levels of IL-1β, IL-6, and TNF-α in serum were markedly elevated in AOM/DSS-induced mice, relative to that in the control group. High levels of IL-1β, IL-6, and TNF-α in serum were significantly inhibited by LFs treatment. Moreover, we observed that LFs treatment could dramatically decrease mRNA levels of IL-1β, IL-6, and TNF-α in colonic tissues at either 50 or 100 mg/kg (Figure 4B).

Next, inducible nitric oxide synthase (iNOS) and cyclooxygenase-2 (Cox-2) expression, as reliable indicators of mucosal inflammation, was examined using qRT-PCR and IHC staining. High mRNA levels of iNOS and Cox-2 was observed in AOM/DSS-induced mice which was substantially alleviated by LFs treatment (Figure 4C). IHC evaluation showed that the expression of Cox-2 in the AOM/DSS treated colons was significantly reduced after treated with LFs (Figure 4D). These data corroborate that LFs treatment attenuate production of pro-inflammatory cytokines and mediators in AOM/DSS induced mice.

### 2.5. LFs Treatment Attenuated Phosphorylation of Jak2 and Stat3

**Figure 5:**
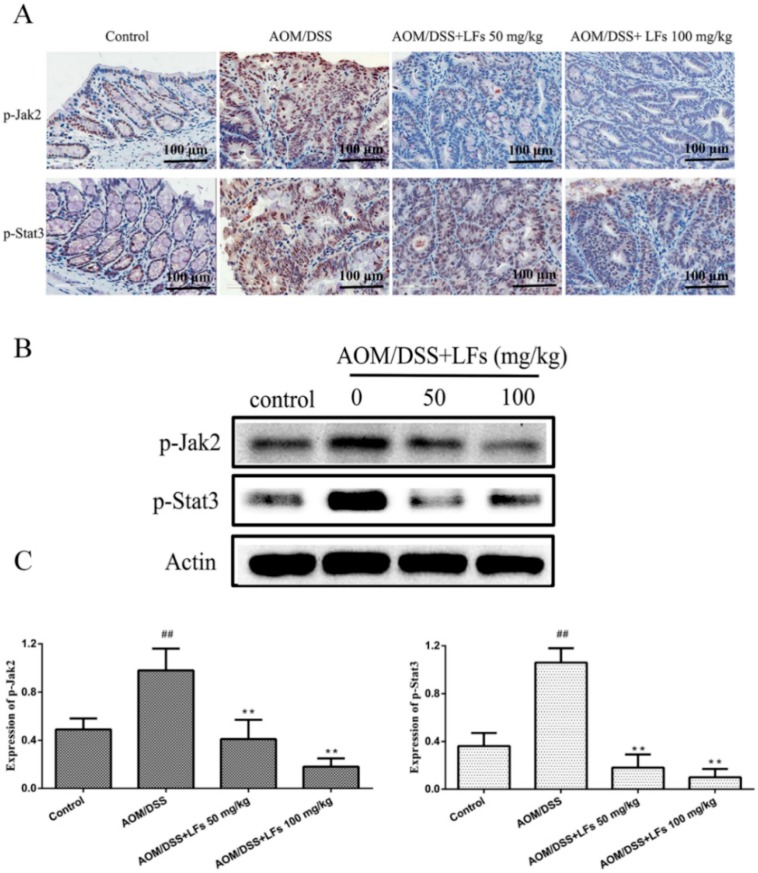
Effects of LFs on phosphorylation of Jak2 and Stat3: (**A**) Immunohistochemical staining of p-Jak2 and p-Stat3 in colonic tissues; and (**B**,**C**) Western blot of p-Jak2 and p-Stat3 expression in colonic tissues and semi-quantitative analysis of these proteins. Data are presented as mean ± SD. ** *p* < 0.01 vs. model, ^##^ *p* < 0.01 vs. vehicle control.

To further elucidate the molecular mechanism underlying the anti-tumor effect of LFs, Western blot and IHC analysis were conducted to examine phosphorylation of oncogenic proteins Jak2 and Stat3, well known as downstream of IL-6, which were reported to play critical roles in the progression of CAC using Western blot and IHC analysis. Figure 5A shows representative sections of the expression of p-Jak2 and p-Stat3. The IHC images showed that expression of p-Jak2 and p-Stat3 were elevated in the colon tissues of mice treated with AOM/DSS, which was markedly decreased after mice were treated with LFs. Moreover, Western blot analyses were consistent with IHC evaluation (Figure 5B,C), verifying that high expression of p-Jak2 and p-Stat3 induced by AOM/DSS was substantially suppressed by LFs treatment.

### 2.6. LFs Inhibited Activation of Nfκb

**Figure 6:**
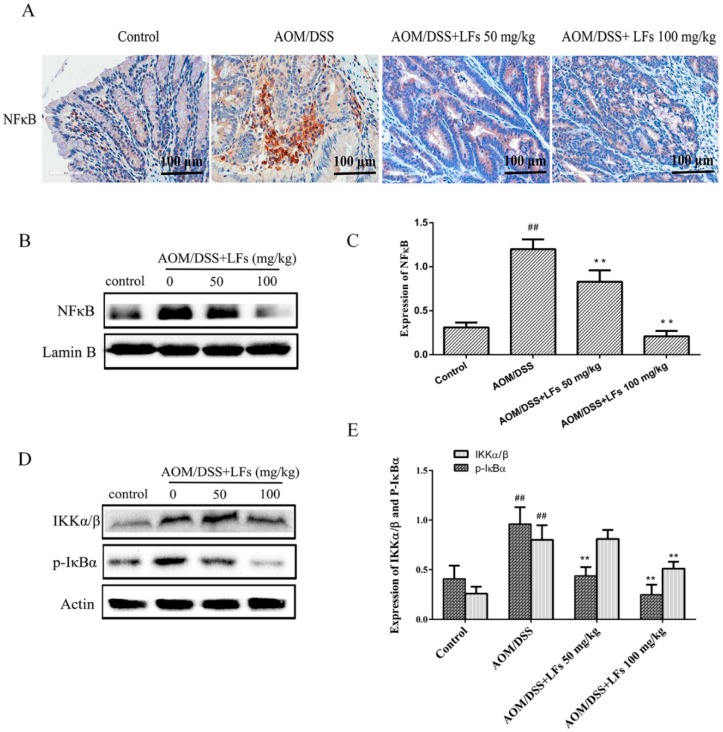
Effects of LFs on NFκB activation. (**A**) Immunohistochemical staining of NFκB in colonic tissues; (**B**,**C**) Nuclear translocation of NFκB assessed by Western blot and semi-quantitative analysis of these proteins. Nuclear proteins were used to conduct Western blot analysis. Data are presented as mean ± SD. ** *p* < 0.01 vs. model, ^##^ *p* < 0.01 vs. vehicle control; (**D**,**E**) Western blot of IKKα/β and p-IκBα expression in colonic tissues and semi-quantitative analysis of these proteins. Data are presented as mean ± SD. ** *p* < 0.01 vs. model, ^##^ *p* < 0.01 vs. vehicle control.

Since nuclear factor-κB (NFκB) activation plays an essential role in modulation of inflammatory cytokines and mediators, we next investigated the activation of NFκB in colon tissues to understand whether NFκB involve in LFs treatment. Initially, NFκB was assessed by IHC analysis, and data in Figure 6A showed that expression of NFκB was markedly elevated in AOM/DSS induced colons, and was substantially decreased by LFs treatment. Western blot analysis showed that LFs significantly reduced nuclear translocation of NFκB (Figure 6B,C).

We next explore expression of IKKα/β and p-IκBα in colon tissues. As shown in Figure 6D,E, LFs significantly decreased the expression levels of IKKα/β and p-IκBα. All these results indicated that LFs inhibit CAC progression through modulating NFκB activation. Taken together, these data suggest that LFs decreased activation of NFκB, thus suppressing expression of pro-inflammatory cytokines and mediators including IL-1β, IL-6, TNF-α, iNOS and Cox-2 and reducing phosphorylation of Jak2 and Stat3, followed by blockage of inflammation and tumorigenesis.

## 3. Discussion

CAC is a malignancy of colon that is strongly linked to IBD [18]. It has been verified that blocking inflammatory pathways is very effective in preventing colonic tumors and their malignant progression developing in both animal and human studies [19,20]. Our previous studies demonstrated that LFs could significantly alleviate inflammation in DSS induced UC [17]; we therefore proposed that LFs may be potent in suppression of AOM/DSS-induced CAC. Here, we assessed the effect of LFs in the suppression of AOM/DSS-induced colitis and colorectal tumorigenesis, and further investigated the underlying mechanisms. Our data provide direct demonstration that oral administration of LFs significantly inhibit tumorigenesis in AOM/DSS-induced colons, which is associated with a marked suppression of NFκB and reduced production of pro-inflammatory mediators (iNOS, Cox-2, IL-1, IL-6, and TNF-α), a response linked to suppression of p-Jak2 and p-Stat3. The tumor suppressor P53 and the corresponding downstream proteins were also altered by treatment with LFs. We show, for the first time, that LFs has potential in CAC treatment, through modulating P53 and NFκB/IL-6/Jak2/Stat3 signaling pathway.

The AOM/DSS induced neoplasm resembles human CAC recapitulating many of the clinical observations associated with CAC in humans [21]. AOM exerts colonic carcinogenicity via causing formation of O6-methylguanine upon metallic activation; meanwhile, cycles of DSS treatment induces chronic inflammation in colons, which mimics IBD [22]. In our research, a single injection of AOM in combination with three cycles of DSS resulted in 100% incidence of colonic neoplasms. Tumor growth and mass death induced by AOM/DSS were markedly reversed by LFs treatment, which was also accompanied by a reduction of the mucosal inflammatory process with a decreased mucosal thickening, suggesting an effective inhibition of tumorigenesis and inflammation.

As one of the first oncogenes activated in carcinogenesis, P53, is a well-established tumor suppressor that plays a prominent role in the progression of CAC [23,24]. During carcinogenesis, P53 acts consistently as a negative regulator of cell proliferation by inducing cell cycle arrest via activating P21WAf1/Cip1 [25]. P21WAf1/Cip1 then functions as a blocker of CDKs which are known to active in late G1, S, G2 and M phases, thus leading to cell cycle arrest [26]. In addition, activated P53 induces pro-apoptotic factors such as Bax leading to increased apoptosis [27]. Here, we show that in mice with CAC, LFs suppresses colonic tumor formation in association with increased apoptosis and cell cycle arrests, as indicated by increased po-apoptotic protein Bax, decreased anti-apoptotic protein Bcl-2, elevated p-P53 and P21, and reduced CyclinD1. These findings suggest that LFs protects against CAC by modulating P53 responses, which are associated with apoptosis and cell cycle arrests.

Current treatments for IBD include inhibition of inflammation by corresponding drugs, because it has been demonstrated that pro-inflammatory cytokines and mediators play a crucial role in the pathogenesis of IBD and CAC, such as IL-1β, IL-6, TNF-α, iNOS and Cox-2 [4,21,28]. Among these cytokines and mediators, there is strong evidence for the role played by IL-6, whose effects are mediated through activation of NFκB, thus leading to recruitment and subsequent phosphorylation of the Jak2 and Stat3 [2,3]. In tumor cells, activation of Stat3 involve in regulation of anti-apoptotic genes and cell cycle regulators, such as Bcl-xL, Bcl-2, Cyclin D1 and PCNA [29,30]. Thus, aberrant NFκB/IL-6/Jak2/Stat3 pathway has been identified recently as a principal signaling associated with progression of IBD and CAC [31]. Consistent with previous studies, here, we observed that AOM/DSS administration invariably promote inflammation in the colon with elevated levels of IL-1β, IL-6, TNF-α, iNOS and Cox-2 and an increased expression of p-Jak2 and p-Stat3. In DSS/AOM-induced mice, the observed up-regulation of pro-inflammatory cytokines, including IL-6, was ameliorated by LFs treatment; meanwhile, phosphorylation of Jak2 and Stat3 was blocked. These results provide further evidence of the effect of LFs in suppression of colitis and tumorigenesis and point to the key role of NFκB/IL-6/Jak2/Stat3 pathway as relevant target of LFs.

It is well established that pro-inflammatory cytokines and mediators, including IL-1β, IL-6, TNF-α, iNOS and Cox-2 which acts as tumor promoter is tightly modulated by transcription factor NFκB [32]. NFκB is previously shown to exist in the cytoplasm in an inactive form held in check by the inhibitor IκB. Degradation of IκB is associated with its phosphorylation induced by the IκB kinase (IKK) complex, namely IKKα-IKKβ-IKKγ. Phosphorylation of IκB then initiates its polyubiquitination and degradation, thus leading to the release and nuclear translocation of NFκB to activate various target including pro-inflammatory mediators and cytokines [33]. Here, we found that exposure of AOM/DSS induced mice to LFs resulted in decreased level of IKKα/β and reduced phosphorylation of IκBα, and thus suppressing nuclear translocation of NFκB. These observations suggests involvement of NFκB in LFs treatment, as a central regulator of pro-inflammatory cytokine and mediator.

In summary, we report that LFs inhibited experimental CAC, resulting in overall attenuation of colitis and tumorigenesis, including reversing reduced colon length and elevated tumor weight to length ratio, reducing tumor numbers, altering the body weight changes, and improving survival rates of AOM/DSS induced mice. These effects were observed together with a significant inhibition of P53 and NFκB/IL-6/Jak2/Stat3 pathway. Thus, this research creates a rationale for the use of LFs in the treatment and prevention of CAC.

## 4. Materials and Methods

Licorice flavonoids (LFs) used in the present study were isolated from *Glycyrrhizain flata* Bat by our group and were identified as a mixture of Licochalcone A, Tetrahydroxychalcone, Echinatin, Formononetin, Pinocembrinchalcone, Licochalcone D, Licoflavone, Glabrone, Licoflavone C, Licochalcone C, Enoxolone, Licoflavone B and Kanzonol E by HPLC, UV, IR, NMR and MS method (Figure S1 and Tables S1 and S2).

### 4.1. Reagents

DSS was purchased from MP Biomedicals (Santa Ana, CA, USA). AOM was purchased from Sigma Aldrich (St. Louis, MO, USA). Phospho-Jak2 (Tyr1007) (D15E2), Phospho-Stat3 (Tyr705) (D3A7), Phospho-IκBα (Ser32) (14D4), Phospho-P53 (Ser15), β-actin and all secondary antibodies were purchased from Cell Signaling Technology (Danvers, MA, USA). NFκB p65 (F-6), IKKα/β (H-470), Bcl-2, Bax, P21 (H-250), Cox2 (H-62) and Cyclin D1 (H-250) were purchased from Santa Cruz Biotechnology (Santa Cruz, CA, USA). Mouse Interleukin-6 (IL-6), Interleukin-1β (IL-1β) and Tumor Necrosis Factor-α (TNF-α) ELISA Kits were products of R&D Systems (Minneapolis, MN, USA).

### 4.2. Animals

Female C57BL/6 mice weighing 16–18 g were obtained from Vital River Laboratory Animal Technology Co., Ltd. (Beijing, China). Mice were housed under standard conditions of temperature and humidity with freedom to water and food, and were subjected to a 12-h light/dark cycle. All mice were used in accordance with protocols approved on 6 November 2015 (No. 15-11-06) by the Animal Ethics Committee at the Institute of Medicinal Plant Development, Chinese Academy of Medical Sciences.

### 4.3. AOM/DSS-Induced Colitis-Associated Carcinogenesis

CAC was induced in mice using AOM and DSS as previously described [34]. Figure 1A outlines the inducement of this model; briefly, mice received a single intraperitoneal (i.p.) injection of AOM (10 mg/kg). One week later, they were exposed to 2.5% DSS (wt/vol) in drinking water for 7 consecutive days followed by 14 days of regular drinking water. This cycle was then repeated twice. Body weight was assessed once a week throughout the course of the experiment.

AOM/DSS-induced mice were gavaged with LFs (0, 50 and 100 mg/kg) once a day starting at the day 1 until the end of the experiment (Figure 1A). Mice in the control group were gavaged with saline. All mice were sacrificed by cervical dislocation at the end of 10th week, the colons were excised from the ileocecal junction to anus, the length and weight of colons were measured, after which the colons were examined macroscopically under a dissecting microscope and tumor numbers were counted. The colons were then either used for histopathology and immunohistochemical (IHC) evaluation by forming “Swiss rolls” and fixing in 10% phosphate-buffered formalin, or for qRT-PCR and Western blot analysis after they were flash-frozen in liquid nitrogen.

### 4.4. Histopathological Analysis

Formalin-preserved colons were dehydrated and embedded in paraffin by standard techniques. Subsequently, paraffin embedded samples were sectioned at 5 µm, stained with hematoxylin and eosin (H&E) and examined by a pathologist blinded to the experimental groups.

### 4.5. Immunohistochemical (IHC) Analysis

Paraffin embedded slides were deparaffinized, rehydrated, and pre-treated with 1% hydrogen peroxidase in PBS buffer to quench the endogenous peroxidase. After blocking with the appropriate antisera, slides were incubated with primary antibodies (1:100) in PBS containing 1% BSA at 4 °C overnight, followed by washing with PBS for three times. Biotinylated secondary antibodies were added and incubated at room temperature for 1 hr. Signals were detected using Diaminobenzidine Substrate kit (Vector Laboratories, Burlingame, CA, USA). The Image-ProPlus 4.5 Software (Media Cybernetics, Bethesda, MD, USA) was used to analyze the expression of proteins.

### 4.6. Measurement of Pro-Inflammatory Cytokines (IL-1β, IL-6, and TNF-α)

IL-1β, IL-6 and TNF-α levels in the serum were measured using mouse ELISA kits specific for each cytokine following the manufacturer’s instructions. All experiments were performed in triplicate.

### 4.7. RNA Isolation and Quantitative Real Time Polymerase Chain Reaction (qRT-PCR) Analysis

Colon tissue samples were frozen in liquid nitrogen and mechanically dissociated in RNA buffer. Total RNA was then extracted using Trizol reagent (Invitrogen, Carlsbad, CA, USA). Reverse transcriptase PCR was performed with 1 µg of total RNA and a Superscript First-Strand Synthesis kit (Aidlad, Beijing, China) for cDNA transcription. qRT-PCR was performed using FastSYBR^®^ Green PCR Master Mix kit (Cowin Biotech Co., Ltd., Beijing, China) at the following thermal conditions: 95 °C for 10 min and 40 cycles at 95 °C for 5 s and 60 °C for 32 s. The following primers were used: TNF-α (Forward: 5′-AGGGTCTGGGCCATAGAACT-3′; Reverse: 5′-CCACCACGCTCTTCTGTCTAC-3′); IL-6 (Forward: 5′-CTCTGCAAGAGACTTCCATCCAGT-3′; Reverse: 5′-GAAGTAGGGAAGGCCGTGG-3′); IL-1β (Forward: 5′-GCCCATCCTCTGTGACTCAT-3′; Reverse: 5′-AAGGCCACAGGTATTTTGTCG-3′); iNOS (Forward: 5′-AGGGAATCTTGGAGCGAGTTG-3′; Reverse: 5′-AGTAGCTGCCGCTCTCATC-3′); Cox-2 (Forward: 5′-CATTCTTTGCCCAGCACTTC-3′; Reverse: 5′-GGCGCAGTTTATGTTGTCTG-3′); GAPDH was used as a reference gene (Forward: 5′-TTGATGGCAACAATCTCCAC-3′; Reverse: 5′-CGTCCCGTAGACAAAATGGT-3′). Real-time PCR data were analyzed based on the 2^−ΔΔ*C*t^ method (Δ*C*_t_ = *C*_t_ (cytokines) − *C*_t_ (GAPDH)) [24], by using GAPDH for normalization. All samples were performed in triplicate for three times.

### 4.8. Western Blot Analysis

Briefly, colon tissues were homogenized in a standard RIPA buffer supplemented with a cocktail of protease and phosphatase inhibitors. Cytoplasmic and nuclear proteins were prepared using a commercial kit (Beyotime Institute of Biotechnology, Shanghai, China) according to manufacturer’s instructions. Samples were then centrifuged at 15,000× *g* for 10 min at 4 °C. Protein concentrations were determined by modified Bradford assay. Equal amount of sample Lysates were separated on a 12% SDS-polyacrylamide gel and proteins were transferred to PVDF membranes. The membranes were then blocked in TBST buffer containing 5% skim milk for 1 h at room temperature and probed with primary antibodies (1:1000) at 4 °C overnight, followed by incubation with horseradish peroxidase (HRP) conjugated secondary antibodies (1:2000). Membranes were detected by enhanced chemiluminescence method using a commercial ECL kit, according to manufacturer’s instructions. The levels of protein expression were quantified by image J software (National Institutes of Health, Bethesda, MD, USA) and normalized to the relative internal standards. All of the experiments were performed in triplicate.

### 4.9. Statistical Analysis

All results are expressed as Mean ± SD where applicable. GraphPad Prism 6.0 software (GraphPad Software, San Diego, CA, USA) was used for statistical analysis. The statistical significance of group differences was analyzed with one-way ANOVA followed by Tukey’s test or Newman–Kueuls test. *p* < 0.05 was considered statistically significant.

## Supplementary Materials

Supplementary materials can be found at www.mdpi.com/1422-0067/17/9/1343/s1.
