# Supplementary material for: Flavonoids Extracted from Licorice Prevents Colitis-Associated Carcinogenesis in AOM/DSS Mouse Model
Source: Int J Mol Sci. 2016 Aug 24;17(9):1343. doi: 10.3390/ijms17091343 (PMC5037654; doi:10.3390/ijms17091343)
Supplement: Supplementary file 1 [file ijms-17-01343-s001.pdf]

Supplementary Materials: Flavonoids Extracted from Licorice Prevents Colitis-Associated Carcinogenesis in AOM/DSS Mouse Model

Xiaowei Huo, Dongyu Liu, Li Gao, Liyong Li and Li Cao

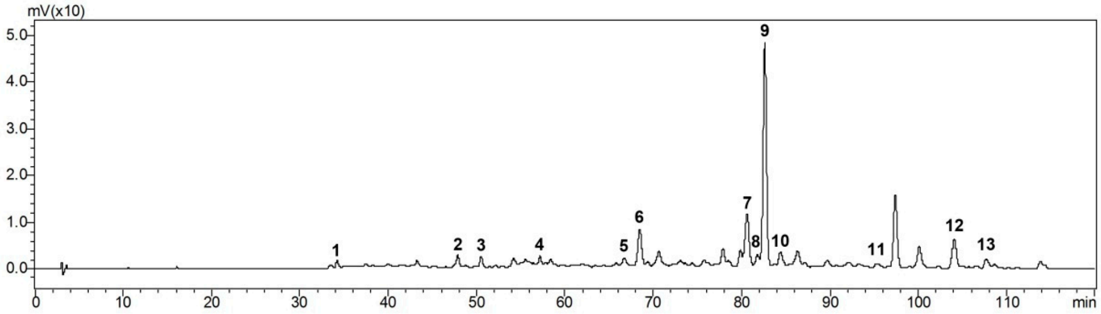

Figure S1. High performance liquid chromatograph of LFs. **1**: Tetrahydroxychalcone; **2**: Echinatin; **3**: Formonoetin; **4**: Pinocembrinchalcone; **5**: Licochalcone D; **6**: Licoflavone; **7**: Glabrone; **8**: Licoflavone C; **9**: Licochalcone A; **10**: Licochalcone C; **11**: Enoxolone; **12**: Kanzonol E; **13**: Licoflavone B.

Table S1. Gradient eluted program of HPLC. A: acetonitrile; B: water with 0.1% formic acid.

Time (min)	Proportion of Mobile Phase (A%)	Proportion of Mobile Phase (B%)
0–10	15–20	85–80
10–12	20–23	80–77
12–17	23–24	77–76
17–30	24–29	76–71
30–40	29–34	71–66
40–50	34–38	66–62
50–50.01	38–42	62–58
50.01–60	42–42	58–58
60–80	42–50	58–50
80–90	50–53	50–47
90–120	53–66	47–34

Table S2. Characterization of compounds in LFs by HPLC.

Peak No.	Compound Name	Molecular Formula	Content (%)	Pub Chem CID
1	Tetrahydroxychalcone	$C_{15}H_{12}O_5$	0.7395	5281222
2	Echinatin	$C_{16}H_{14}O_4$	1.3792	6442675
3	Formononetin	$C_{16}H_{12}O_4$	0.9629	5280378
4	Pinocembrin chalcone	$C_{15}H_{12}O_4$	0.9310	6474295
5	Licochalcone D	$C_{21}H_{22}O_5$	1.4441	10473311
6	Licoflavone	$C_{20}H_{20}O_5$	4.9697	14218027
7	Glabrone	$C_{20}H_{16}O_5$	6.9480	5317652
8	Licoflavone C	$C_{20}H_{18}O_5$	1.5472	10246505
9	Licochalcone A	$C_{21}H_{22}O_4$	23.2111	5318998
10	Licochalcone C	$C_{21}H_{22}O_4$	0.5787	9840805
11	Enoxolone	$C_{30}H_{46}O_4$	0.7123	10114
12	Licoflavone B	$C_{25}H_{26}O_4$	4.3426	11349817
13	kanzonol E	$C_{25}H_{24}O_4$	0.6210	15516846
